# The TRAF2-p62 axis promotes proliferation and survival of liver cancer by activating mTORC1 pathway

**DOI:** 10.1038/s41418-023-01164-7

**Published:** 2023-04-20

**Authors:** Xue Liang, Jiping Yao, Danrui Cui, Weiyang Zheng, Yanning Liu, Guohua Lou, Bingjue Ye, Liyan Shui, Yi Sun, Yongchao Zhao, Min Zheng

**Affiliations:** 1 State Key Laboratory for Diagnosis and Treatment of Infectious Diseases, National Clinical Research Centre for Infectious Diseases, Collaborative Innovation Centre for Diagnosis and Treatment of Infectious Diseases, The First Affiliated Hospital, Zhejiang University School of Medicine, Hangzhou, Zhejiang Province 310003 China; 2 Department of Hepatobiliary and Pancreatic Surgery, the First Affiliated Hospital, Zhejiang University School of Medicine, Hangzhou, Zhejiang Province 310003 China; 3 Cancer Center, Zhejiang University, Hangzhou, China; 4 Institute of Translational Medicine, Zhejiang University School of Medicine, Hangzhou, 310029 China; 5 Cancer Institute of the Second Affiliated Hospital and Institute of Translational Medicine, Zhejiang University School of Medicine, Hangzhou, 310029 China

**Keywords:** Ubiquitylation, Ubiquitin ligases

## Abstract

TRAF2 (Tumor necrosis factor receptor-associated factor 2) is a dual function protein, acting as an adaptor protein and a ubiquitin E3 ligase, which plays an essential role in mediating the TNFα-NFκB signal pathway. Dysregulated expression of TRAF2 has been reported in a variety of human cancers. Whether and how TRAF2 regulates the growth of liver cancer cells remains elusive. The goal of this study is to investigate potential dysregulation of TRAF2 and its biological function in liver cancer, and to elucidate the underlying mechanism, leading to validation of TRAF2 as an attractive liver cancer target. Here, we reported TRAF2 is up-regulated in human liver cancer cell lines and tissues, and high TRAF2 expression is associated with a poor prognosis of HCC patients. Proteomics profiling along with Co-immunoprecipitation analysis revealed that p62 is a new substrate of TRAF2, which is subjected to TRAF2-induced polyubiquitination via the K63 linkage at the K420 residue. A strong negative correlation was found between the protein levels of p62 and TRAF2 in human HCC samples. TRAF2 depletion inhibited growth and survival of liver cancer cells both in vitro and in vivo by causing p62 accumulation, which is partially rescued by simultaneous p62 knockdown. Mechanistically, TRAF2-mediated p62 polyubiquitylation activates the mTORC1 by forming the p62-mTORC1-Rag complex, which facilitates the lysosome localization of mTORC1. TRAF2 depletion inhibited mTORC1 activity through the disruption of interaction between p62 and the mTORC1 complex. In conclusion, our study provides the proof-of-concept evidence that TRAF2 is a valid target for liver cancer.

## Introduction

Hepatocellular carcinoma (HCC) is the fourth leading cause of cancer death globally [1]. The exact mechanism leading to the occurrence and development of HCC still remains unclear. Researches have shown that several significant cellular signaling pathways, including the ubiquitin-proteasome system, nuclear factor κB (NF-κB) pathway, autophagy pathway, among others, are involved in HCC development [2–4]. Studies of the pivotal molecules in these signaling pathways may elucidate the mechanisms of liver tumorigenesis for target discovery, validation, and eventually the development of targeted therapy for effective treatment of HCC.

Tumor necrosis factor receptor-associated factor 2 (TRAF2) is one of the most studied members of the TRAF family and is well known for its scaffolding functions in NF-κB signaling pathways. Thus, TRAF2 plays a significant role in a variety of biological processes, including cellular proliferation, differentiation, and apoptosis [5–7]. In addition to being a signal hub, TRAF2 is also an ubiquitin E3 ligase. TRAF2 mediates both the K63 and K48 polyubiquitin chains of substrates, leading to the activation of downstream signaling [8–10]. *TRAF2* is frequently amplified and rearranged in 15% of epithelial tumors, including HCC, and is identified to be an oncogene [11]. Additionally, TRAF2 also plays a specific role in hematological malignancies [12–14]. Although TRAF2 is involved in a variety of tumors, its role in liver cancer remains elusive. Nevertheless, a recent study reported that combined genetic deletion of receptor-interacting serine/threonine-protein kinase 1 (RIPK1) and TRAF2 in liver parenchymal cells promoted the development of HCC [15], suggesting an active role of TRAF2. In this study, we sought to identify potential proteins interacting with TRAF2 to elucidate the mechanism by which TRAF2 regulates growth and survival of liver cancer cells. This effort led to the discovery of p62 as a candidate.

p62/SQSTM1 (sequestosome 1) is a notable autophagy substrate and adaptor via interacting with microtubule-associated protein light chain 3 (LC3) [16]. In addition to its role in the autophagy pathway, p62 is also a multifunctional signaling regulator involved in cell death, proliferation, and oxidative stress response [17]. Increasing lines of evidence suggest that p62 plays a crucial promoting role in liver tumorigenesis through activating NF-κB signal, and regulating Keap1-NRF2 and mTOR signal pathways, as well as autophagy [18]. In contrast to its oncogenic effect, the loss of p62 in hepatocytes was found to enhance liver tumorigenesis induced by diethylnitrosamine (DEN) in combination of HFD (high-fat diet) [19]. Thus, it appears that p62 may play a complex role in HCC development in a context-dependent manner.

Here, we reported that TRAF2 expression is significantly increased in human HCC tissues at both protein and mRNA levels, which is positively correlated with HCC progression and poor survival of patients. TRAF2 promotes the growth of liver cancer cells both in in vitro and in vivo. Mechanically, we identified that p62 is a new substrate of TRAF2, which is subjected to TRAF2-induced polyubiquitination via the K63 linkage on K420 residues. The ubiquitinated p62 then activates mTORC1 signal via enhancing the lysosome location of mTORC1. Thus, our study suggests a potential strategy by targeting the TRAF2/p62/mTOR axis for the treatment of HCC.

## Results

### High TRAF2 expression is associated with poor prognosis of HCC patients

**Fig. 1:**
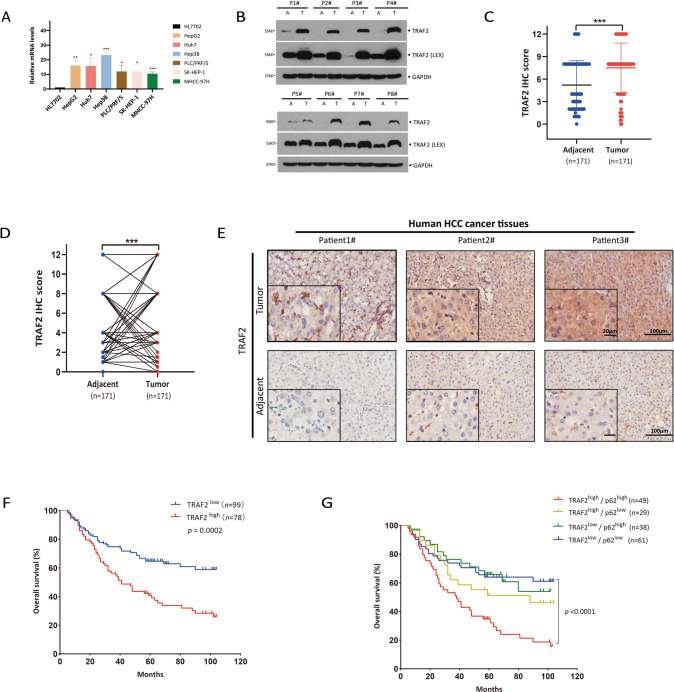
High TRAF2 expression is associated with poor prognosis of HCC patients. **A** The expression of TRAF2 mRNA level in HL7702 liver cell and HCC cell lines was determined by qPCR. Levels of significance: *p < 0.05, **p < 0.01, ***p < 0.001, (Student’s *t*-test). **B** TRAF2 protein levels in eight paired HCC clinical samples were detected by western blot. T: tumor tissue, A: adjacent non-tumor tissue. **C**, **D** HCC tissue microarrays containing 171 tumor tissues and corresponding tumor-adjacent normal tissues were stained for TRAF2 expression. IHC score was calculated via multiplying the percentage score by the intensity score [52, 53]. (***p < 0.001, Student’s *t*-test, two-tailed, mean ± SEM). **E** Representative images from immunohistochemical staining of TRAF2, Scale bar = 20 μm. **F** The correlation between TRAF2 expression and overall survival in HCC patients (*p* = 0.0002, Kaplan-Meier survival analysis). **G** The correlation between TRAF2 and p62 expression and overall survival of HCC patients (*p* < 0.0001, Kaplan-Meier survival analysis).

Our previous study showed that TRAF2 promoted the growth of glioblastoma and lung cancer cells [20]. However, the role of TRAF2 in liver cancer is unknown. To this end, we first evaluated TRAF2 gene expression in liver cancer cell lines and HCC tissues. Quantitative polymerase chain reaction (qPCR) showed TRAF2 mRNA levels were increased both in HCC cell lines and HCC clinical tissues compared to normal liver HL7702 cells (Fig. 1A) and adjacent non-tumor tissues, respectively (Fig. S1B). An elevated TRAF2 protein levels were also confirmed by western blot and immunohistochemistry staining of HCC tissue microarray, consisting of 171 paired HCC samples and corresponding adjacent non-tumor tissues, based on the IHC staining intensity score (Figs. 1B, C, D, E, S1A). Moreover, TRAF2 protein levels in HCC were prominently associated with the clinicopathological parameters such as the tumor grade (Supplementary Table).

Significantly, the survival analysis revealed that HCC patients with high TRAF2 expression tend to have worse overall survival rates than those with lower TRAF2 expression (Fig. 1F). The Cancer Genome Atlas (TCGA) database mining also showed that high TRAF2 expression is correlated with worse prognosis of HCC patients, consistent with our own observation (Fig. S1C). In addition, we analyzed the correlation between TRAF2 and p62 expression and prognosis of HCC patients. Significantly, we found that patients with high TRAF2 and p62 expression had a significantly worse overall prognosis (Fig. 1G). Together, these results demonstrate that TRAF2 was highly expressed at both protein and mRNA levels in HCC tissues, and higher TRAF2 expression predicted a worse prognosis of HCC patients, indicating that increased TRAF2 expression might play a role in HCC tumorigenesis.

### TRAF2 binds to p62

**Fig. 2:**
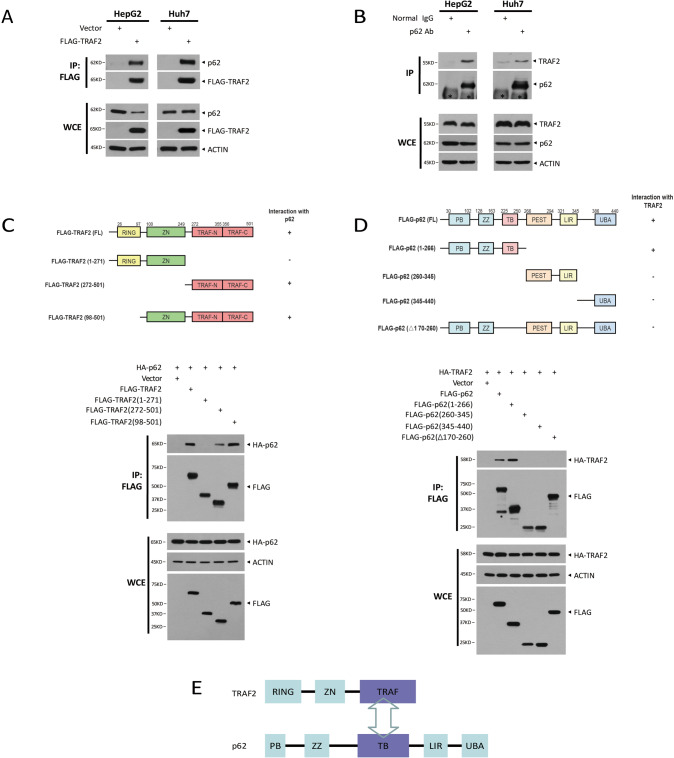
TRAF2 binds to p62. **A** Exogenous interaction between TRAF2 and p62 in HepG2 and HuH7 cells. WCE: whole-cell extracts. **B** Endogenous interaction of TRAF2 and p62. *: IgG heavy chain. **C** A schematic representation of TRAF2 deletion mutants(up), and the interaction between p62 and TRAF2 truncations (below). **D** Mapping of p62 regions interaction with TRAF2 (up), and the interaction of TRAF2 with p62 mutants (below). *: non-specific band. **E** The binding model of TRAF2 with p62.

TRAF2 is an important signal protein involved in important biological processes such as cell growth and differentiation via interaction with a variety of substrates or adaptor proteins. To explore the potential partners of TRAF2 by which TRAF2 promotes the proliferation of liver cancer cells, we generated stable lines in HepG2 and Huh7 cells, expressing FLAG-tagged TRAF2. By tandem affinity purification and mass spectrometry analysis, we identified p62 as a candidate of TRAF2 binding protein (Fig. S2A). To confirm the interaction of TRAF2 with p62, we performed the immunoprecipitation (IP) in HepG2 and Huh7 cells with stable expression of FLAG-TRAF2. Indeed, p62 was detected in immunoprecipitates pulled down by the FLAG antibody (Fig. 2A). We further confirmed the TRAF2-p62 binding in human 293 and PLC/PRF/5 cells (another HCC cell line), transiently transfected with FLAG-tagged TRAF2 (Fig. S2B). Finally, we examined endogenous binding between TRAF2 and p62. Cell lysates of HepG2 and Huh7 were immunoprecipitated with p62 antibody, and TRAF2 was readily detected (Fig. 2B). Collectively, these results clearly demonstrated that TRAF2 binds to p62.

Previous studies have shown that TRAF2 contains multiple domains, including the N-terminus RING domain, Zing finger domain at the center, and C-terminus TRAF domain, all of which have been shown to mediate the protein-protein interactions [7]. We next mapped the TRAF2 domain(s) which mediates the TRAF2-p62 binding. Three TRAF2 deletion mutants, along with full-length TRAF2 were transiently co-transfected with and HA-tagged p62 plasmids into 293 cells. Cell lysates were immunoprecipiated with anti-FLAG antibody, followed by immunoblotting with anti-HA antibody. The results showed that all the TRAF2 domain-containing constructs bound to p62, whereas the truncated mutant encoding RING-Zn domains failed to bind p62 (Fig. 2C), indicating that the TRAF domain is responsible for p62 binding.

Similarly, p62 also comprises of several conserved domains, contributing to protein-protein interaction [21]. To determine which domain of p62 was required for the TRAF2 association, various p62 deletion mutants with FLAG-tag were generated and co-transfected with HA-TRAF2. The subsequent IP-IB assays showed that only TB domain-containing p62 constructs were able to pull-down TRAF2 (Fig. 2D), demonstrating that TRAF2 bind to p62 via its TB domain. Taken together, the TRAF domain of TRAF2 and TB domain of p62 mediate the TRAF2-p62 binding (Fig. 2E).

### TRAF2 negatively regulates the protein levels of p62 and autophagy flux

**Fig. 3:**
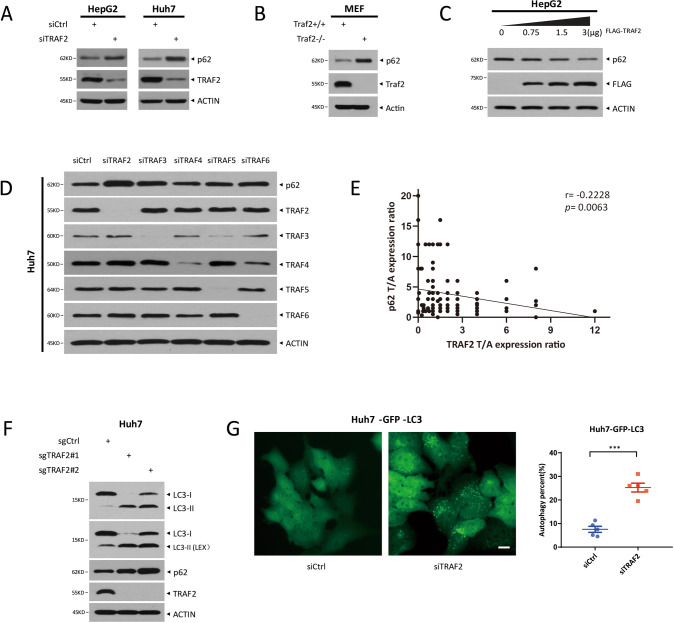
TRAF2 negatively regulates the protein levels of p62 and autophagy flux. **A**, **B** Knockdown or knockout of TRAF2 increases p62 level. **C** TRAF2 overexpression decreases the p62 level. **D** Huh7 was transfected with siCtrl and TRAF family siRNA oligos for 48 h, and p62 protein level was analyzed by Western blot. **E** Correlation of TRAF2 and p62 in HCC tissue microarrays (*p = 0.0063, Pearson’s correlation coefficient). T, tumor tissue. A, adjacent nontumor tissue. **F** LC3 and p62 protein level in TRAF2-deletion Huh7 cells. **G** LC3 punctate structures were shown by fluorescence microscopy (left, bar:10 μm), ***p < 0.001 (right, Student’s *t*-test, two-*t*ailed, mean ± SEM, *n* = 5).

Several studies have revealed that p62 was involved in liver cancer development [22]. To explore the possible effects of TRAF2 on p62, we first investigated whether TRAF2 influenced p62 protein expression in HCC cell lines. Remarkably, TRAF2 knockdown via siRNA silencing approach significantly increased the p62 protein level in both HepG2 and Huh7 cells (Fig. 3A) and similar results were found in other HCC cell lines (Fig. S3A). We then repeated the experiment in paired *Traf2*^-/-^ MEF cells, and consistently, *Traf2* depletion caused p62 accumulation (Fig. 3B). Conversely, TRAF2 overexpression decreased endogenous p62 levels in a dose-dependent manner (Figs. 3C and S3B).

Previous studies reported that p62 interacted with both TRAF6 and TRAF3 [23, 24]. we next compared the effect on p62 levels upon knockdown of TRAF family members and found that only TRAF2 knockdown triggered the highest level of p62 accumulation (Figs. 3D and S3C). Finally, we found a significant negative correlation in the levels of TRAF2 and p62 protein in human HCC samples (Fig. 3E), indicating an inverse relationship of these two proteins.

One of the well-known functions of p62 is to regulate autophagy. Since TRAF2 negatively regulated the p62 levels, we next explored whether TRAF2 had an effect on autophagy. We first constructed HCC cell lines with TRAF2 knockout via CRISPR/Cas9 system and examined the effect on autophagy upon TRAF2 depletion. Significantly, TRAF2 knockout remarkably increased the LC3-II conversion (Fig. 3F), a typical biomarker of autophagy induction. We next examined the role of TRAF2 in regulation of autophagic flux by constructing the GFP-LC3 stable expressing Huh7 cells, followed by transfection of siTRAF2 and siCtrl oligos. We found that TRAF2 knockdown induced a significant increase in autophagosomes (Fig. 3G). Together, these results demonstrated that TRAF2 knockdown disrupts autophagic flux in HCC cells.

### TRAF2 promotes K63-linked polyubiquitination of p62 on 420 lysine residue and lysosome-dependent degradation

**Fig. 4:**
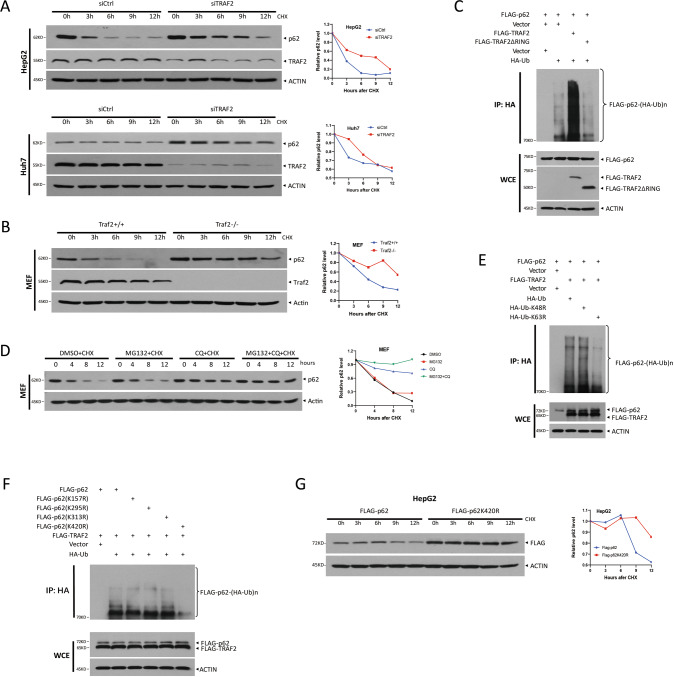
TRAF2 promotes K63-linked polyubiquitination of p62 at K420 residue and lysosome-dependent degradation. **A**, **B** p62 half-life. HCC cells and Traf2-deletion MEF cells were treated with CHX (100 μg/ml) for indicated time periods. **C** TRAF2 promotes ubiquitination of p62 rely on RING domain. **D** MEF cells with CHX (100 μg/ml) were treated with MG132(20 μM), CQ (50 μM), and MG132 with CQ separately for indicated time periods. **E**, **F** TRAF2 promotes K63 polyubiquitination of p62 at K420site. **G** HepG2 cells were transfected with FLAG-tagged p62 plasmid or mutant forms, then treated with CHX (100 μg/ml) for indicated time periods, following IB with FLAG and ACTIN Abs.

In addition to playing an important role in signal transduction, TRAF2 is also a well-established E3 ligase, which activates downstream signaling by promoting the polyubiquitylation of substrate proteins via both K63 and K48 linkage [8–10, 25, 26]. Since we found that TRAF2 interacts with p62 and negatively regulates p62 protein level, we hypothesized that TRAF2 mediates the ubiquitin-proteasome degradation of p62. To test this hypothesis, we first examined whether TRAF2 affects p62 protein half-life by treating cells with cycloheximide to block new protein synthesis. As shown in Fig. 4A, p62 half-life was prolonged up to greater than 9 h by TRAF2 knockdown, while compared to 3 h in the siCtrl group. Likewise, Traf2 half-life was also significantly extended in *Traf2*^-/-^ MEF cells (Fig. 4B).

We further examined whether p62 is subjected to TRAF2-induced polyubiquitylation. To address this, FLAG-p62, together with HA-Ub, FLAG-TRAF2, and FLAG-TRAF2 (ΔRING) mutant were transfected into 293 cells, followed by in vitro ubiquitination assay. Indeed, wild type, but not RING-deleted ligase-dead mutant, TRAF2 significantly promoted p62 polyubiquitylation (Fig. 4C), indicating a ligase-dependent mechanism.

In general, polyubiquitylation via the K48 linkage targets the substrates for degradation, while polyubiquitylation via the K63 linkage often modulate the activity of the substrates, often leading to signaling activation and trafficking [27]. Hence, we wondered whether in our case, TRAF2 might mediate p62 polyubiquitylation via the K48 linkage, since TRAF2 negatively regulated the protein level of p62. We examined the effect of TRAF2 on the protein half-life of p62 in the absence or presence of proteasome inhibitors or autophagy inhibitor, given that p62 degradation through the autophagy pathway is well-known. To our surprise, proteasome inhibitor MG132 had no effect, whereas autophagy inhibitor chloroquine completely blocked TRAF2-induced p62 degradation (Figs. 4D, S4A). The in vivo ubiquitylation assay showed that only the Lys63-ubiquitin mutant (K63R) significantly inhibited the formation of p62 polyubiquitylation chain (Fig. 4E). To further verify that TRAF2 promotes K63-linked polyubiquitination on p62 under physiological settings, we reconstituted TRAF2 (WT) and TRAF2 (ΔRING) mutant constructs in Traf2 knock-out MEF cells, and IP with p62 antibody, followed by IB with K63-linkage specific polyubiquitin antibody. Consistently, wild-type TRAF2, but not ΔRING mutant TRAF2, promotes K63-linked polyubiquitination of p62 (Fig. S4C). Taken together, our results demonstrated that TRAF2 promotes p62 polyubiquitylation via the K63 linkage and p62 degradation is mediated via lysosomal, rather than proteasomal system.

We next investigated which lysine residues are responsible for p62 ubiquitination. Four ubiquitylation sites (K157, K295, K313, and K420) were identified from both endogenous and in vitro p62 ubiquitylation, according to a recent study [28]. Therefore, we established four Lys-ubiquitin mutants and transfected them into 293 cells, and found that the K420R mutant had significantly reduced ubiquitin chains, indicating the K420 is likely the site for p62 polyubiquitination (Fig. 4F). Furthermore, we reconstituted re-transfected p62 WT and K420R constructs in p62 deletion Huh7 cells, and IP with p62 antibody, followed by IB with K63-linkage specific polyubiquitin antibody. We found that the p62 K420R mutant had notably decreased K63-polyubiquitin chains compared to p62 WT, which further demonstrated that K420 is the site for p62 K63-linked polyubiquitination (Fig. S4D). Moreover, we also constructed p62K420R knock-in Huh7 cell via a CRISPR/Cas9-based approach, similarly, we demonstrated the decreased K63-polyubiquitin chains in p62K420R knock-in Huh7 cell (Fig. S4E). Consistently, the half-life of the K420R mutant was much prolonged, compared to WT and other mutants (Figs. 4G, S4B). Collectively, TRAF2 promoted p62 ubiquitination and mediated the K63-linked polyubiquitination of p62 at Lys 420 site.

### TRAF2 promotes growth of liver cancer cells in vitro and in vivo by activating mTORC1

**Fig. 5:**
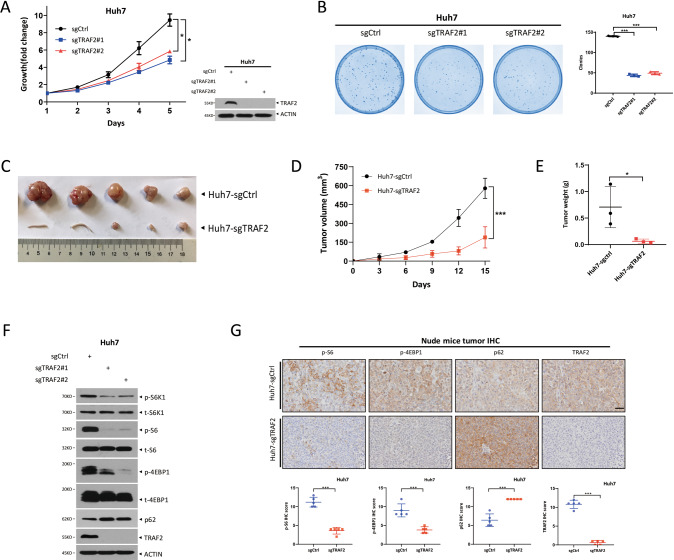
TRAF2 promotes tumor growth in vitro and in vivo by activating mTORC1. **A** TRAF2 deletion inhibits Huh7 cell growth. (*p < 0.05, *n* = 3, two-way repeated-measures ANOVA analysis). **B** Knockout of TRAF2 decreased Huh7 cell colony formation ability. (***p < 0.001, *n* = 3, Student’s *t* test, mean ± SEM). **C**, **D**, and **E** Effects of TRAF2 on nude mice tumor growth. (*n* = 5 for each group, ***p < 0.001, *p < 0.05; **D** two-way repeated-measures ANOVA analysis, **E** Student’s *t* test, mean ± SEM). **F** TRAF2-deletion Huh7 cells showed decreased mTORC1 activity. **G** Representative immunohistochemistry images of nude mice tumor tissues (up), IHC score of indicated protein (below, ***p < 0.001, Student’s *t* test, mean ± SEM), Scale bar = 100 μm.

Given that TRAF2 was highly expressed in human HCC cell lines and tissue samples, it is likely that TRAF2 could play a promoting role in the development of HCC. To determine whether TRAF2 indeed promotes the proliferation and survival of HCC cells, we generated TRAF2-knockout Huh7 and Hep3B cell lines, using CRISPR/Cas9 mediated genome editing system. After puromycin screening, we selected two individual clones from each cell line. The ATPlite-based growth assay showed that TRAF2 knockout in Huh7 and Hep3B cells significantly inhibited cell growth compared with that of sgCtrl cells (Figs. 5A, S5A). Likewise, the clonogenic survival assay showed that TRAF2 depletion remarkably suppressed the colony formation (Figs. 5B, S5B). On the other hand, a gain-of-function study was conducted to determine the effects of TRAF2 overexpression on the proliferation and survival of HCC cells. After re-transfected TRAF2 plasmid into TRAF2-deletion HCC cells, the cell growth and clonogenic survival were much improved, compared to the vector group (Fig. S5C, D).

To further verify the role of TRAF2 in tumor growth in vivo, we established a xenograft model by injecting the paired sgCtrl and sgTRAF2 huh7 cells into nude mice. Indeed, TRAF2 deletion significantly inhibited nude mice tumor formation, with a reduced tumor volume and tumor weight in sgTRAF2 nude mice compared to sgCtrl mice (Fig. 5C, D, E).

Mechanistically, we found that TRAF2 knockout significantly inhibited mTORC1 activity, as evidenced by reduced levels of phosphorylated ribosomal protein S6 kinase 1 (p-S6K1), phosphorylated ribosomal protein S6 (p-S6) and phosphorylated translational initiation factor 4E-binding protein 1 (p-4EBP1) in TRAF2 depletion HCC cell lines and nude mice tumor tissues (Figs. 5F,G, S5E). Thus, TRAF2 is required for tumor formation in nude mice xenograft model with the mechanism associated with mTORC1 signaling pathway.

### TRAF2 promotes tumor growth in vitro and in vivo in a p62-dependent manner

**Fig. 6:**
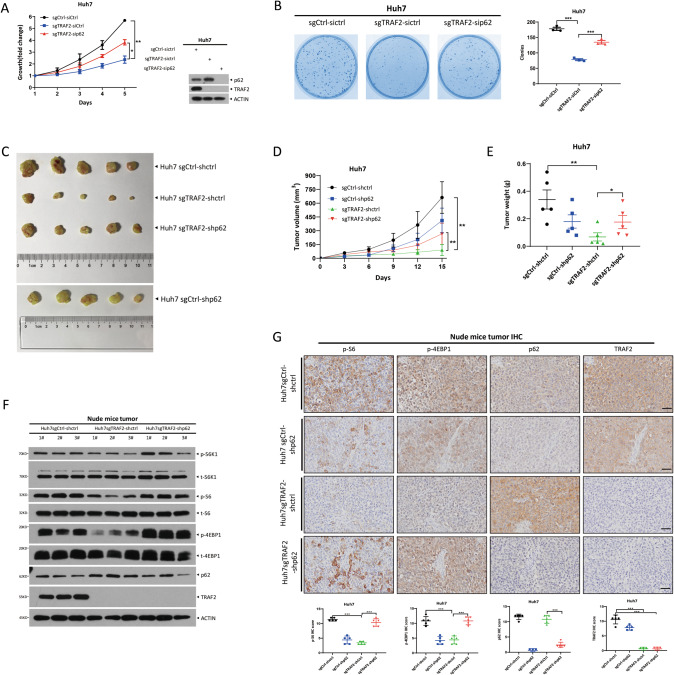
TRAF2 promotes tumor growth in vitro and in vivo in a p62-dependent manner. **A** Cell proliferation was evaluated by the ATPlite growth assay for 5 days (*p < 0.05, **p < 0.01, *n* = 3, two-way repeated-measures ANOVA analysis). **B** Cell clonogenic ability was expressed by coomassie blue staining (left) and colony counting (right) (Student’s *t* test, mean ± SEM, *n* = 3, ***p < 0.001). **C**, **D**/**E** Knockdown of p62 partially rescued TRAF2-mediated tumor suppression, tumor volumes, and weight were measured (*n* = 5 for each group, *p < 0.05, **p < 0.01, **D** two-way repeated-measures ANOVA analysis; **E** Student’s *t* test, mean ± SEM). **F** mTORC1 activity was analyzed in nude mice tumor tissues, following IBs with indicated Abs. **G** Representative immunohistochemistry images (up). IHC score of indicated protein (below, ***p < 0.001, *n* = 5, Student’s *t* test, mean ± SEM). Scale bar = 100 μm.

It is well-established that p62 plays a vital role in liver cancer development through several signal pathways [18]. Since we identified p62 as a substrate of TRAF2, we next determined potential involvement of p62 in the growth and survival of liver cancer cells suppressed by TRAF2 knockdown/knockout. The p62 knockdown in TRAF2-depleted cells with increased p62 level promoted the growth and survival of liver cancer cells (Figs. 6A/B and S6A/B), suggesting that p62 plays a growth-suppressive role under our assay condition.

We then used mouse xenograft tumor model to extend the in vitro cell culture observation to an in vivo setting. Lentivirus with shRNA targeting p62 were transfected into Huh7 cells with TRAF2 depletion, and a stable clone was selected and expanded, followed by inoculation into nude mice. Similarly, p62 knockdown significantly reversed tumor growth inhibition by TRAF2 knockdown with increased tumor volume and weight (Fig. 6C, D, E).

Since TRAF2 knockdown inactivated mTORC1 activity, we finally determined the effect of simultaneous knockdown of p62 and TRAF2, as compared to TRAF2 knockdown alone, on mTORC1 activity. As shown in Fig. 6F/G, p62 knockdown reversed the inactivation of mTORC1 activity by TRAF2 knockdown. In order to clarify that mTORC1 activation is the downstream event responsible for liver cancer growth, we inhibited mTORC1 activation by silencing Raptor, a key component of mTORC1, and generated Huh7-sgTRAF2-shRaptor and Huh7-sgTRAF2-shp62-shRaptor cells. We found that the growth of Huh7-sgTRAF2-shp62-shRaptor, but not Huh7-sgTRAF2-shRaptor cells, was significantly inhibited along with decreased levels of p-S6K1, p-S6, and p-4EBP1, the well-known markers of mTORC1 activity, in vitro cell-based and in vivo nude mice tumor formation-based settings, when compared with each respective control cells (Fig. S6C–H). Taken together, p62 knockdown partially rescued tumor growth suppression both in vitro and in vivo by TRAF2 knockdown, via at least in part, reactivation of mTORC1 activity.

### Ubiquitinated p62 by TRAF2 promotes mTOR activation

**Fig. 7:**
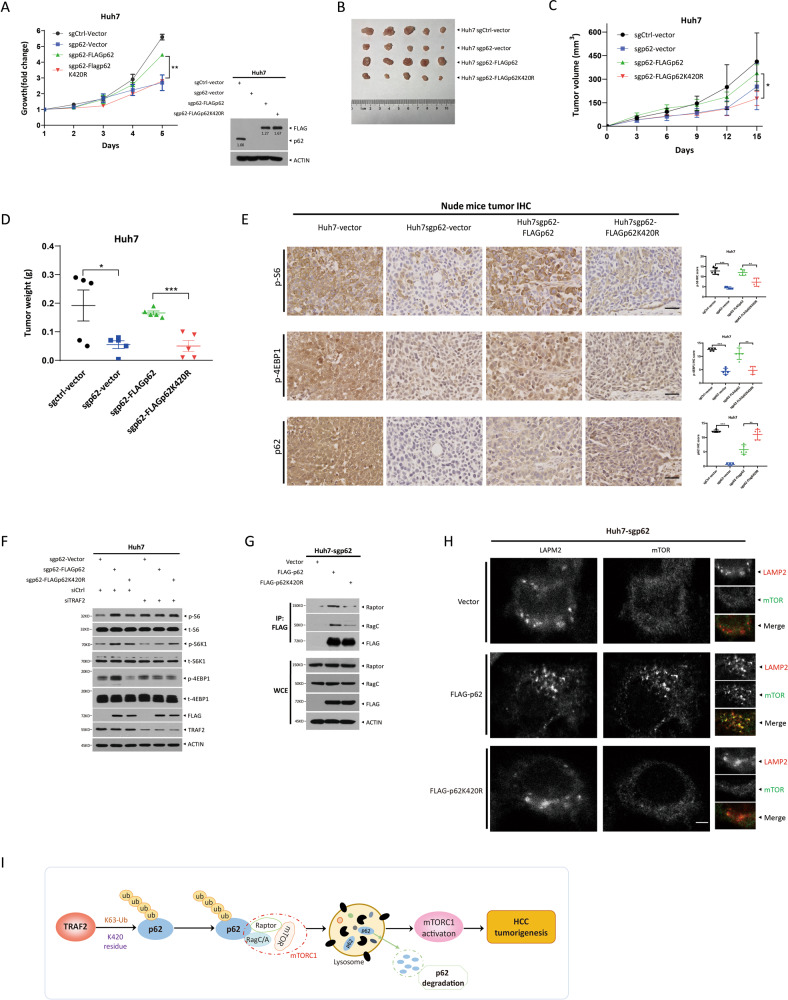
Ubiquitinated p62 by TRAF2 promotes mTOR activation. **A** Cell proliferation was evaluated by ATPlite growth assay for 5 days (**P < 0.01, *n* = 3, two-way repeated-measures ANOVA analysis). **B**, **C**, **D** Ubiquitinated p62 promotes HCC cell proliferation compared to K420R mutants, tumor volumes, and weight were measured (*p < 0.05, ***p < 0.001, *n* = 5, **C** two-way repeated-measures ANOVA analysis; **D** Student’s *t* test, mean ± SEM). **E** Representative immunohistochemistry images(left). IHC score of indicated protein (right, ***p < 0.001, *n* = 5, Student’s *t* test, mean ± SEM). Scale bar = 50 μm. **F** mTORC1 activity was analyzed in Huh7 cell, following IBs with indicated Abs. **G** The interaction of ubiquitinated p62 with Raptor and RagC. **H** Confocal microscopy showing the colocalization of LAMP2 and mTOR in p62-deletion Huh7 cells. Scale bar = 5 μm. **I** A model for TRAF2 in liver tumorigenesis.

Having established that p62 knockdown partially rescued growth suppression both in vitro and in vivo by TRAF2 knockdown, we hypothesized that the ubiquitinated p62 might be a dominant form in HCC. To test this hypothesis, we re-transfected FLAG-tagged p62, along its K420R ubiquitin-dead mutant into p62-depleted Huh7 cells, and established the stable cell line. Reintroduction of WT p62, but not its ubiquitin-dead mutant significantly promoted growth in both cell culture setting (Fig. 7A) and nude mice xenograft model (Fig. 7B–D). Consistently, reintroduction of WT, but not ubiquitin-dead mutant p62 reactivated mTORC1 activity in tumor tissues (Figs. 7E, S7A) as well as in transfected cell lines (Fig. 7F). Moreover, upon mTORC1 inactivation via Raptor knockdown, the growth of reintroduction of WT p62, but not its ubiquitin-dead mutant, is significantly reduced in both cell culture setting (Fig. S7B, C) and nude mice xenograft model, when compared with each respective control group (Fig. S7D–G), suggesting that mTORC1 activation is the downstream event for TRAF2-p62 axis regulating liver cancer growth.

Finally, we investigated how p62 ubiquitination affected mTORC1 activity. A previous study showed that p62 interacts with Raptor, thus as an integral part of the mTORC1 complex, as well as interacts with Rag family proteins for mTORC1 activation [29]. We, therefore, performed the immunoprecipitation experiment to determine whether the ubiquitination status of p62 affected its interaction with Raptor and Rags. Indeed, compared to WT p62, K420R mutant had reduced binding with Raptor and RagC (Fig. 7G). Likewise, the colocalization of mTOR with LAMP2 was decreased in cells expressing K420R mutant, as compared to WT p62 (Fig. 7H), indicating a reduced mTORC1 reactivation. Taken together, it appears that ubiquitinated p62 by TRAF2 is responsible for mTORC1 activation, which further promotes the growth of liver cancer cells.

## Discussion

Liver cancer is one of the most common causes of cancer-related death and is the second most lethal death cancer with a low 5-year survival [30]. Recently, an improved understanding of pivotal genes and related signal pathways have led to several possible therapeutic targets for HCC treatment [31–34]. Thus, molecular characterization of key molecules and the signaling pathways they regulate would lead to identification of therapeutic targets or prognosis biomarkers for effective treatment of this deadly disease.

TRAF2 is an important adaptor protein involved in several signal pathways, especially the NF-κB pathway, and is closely related to cancer development [35–39]. Our previous study showed that TRAF2 is an attractive radio-sensitizing target for glioblastoma and lung cancer [20]. The role of TRAF2 in the development of HCC, however, appears controversial. Shen et al. reported that TRAF2 acts as an NF-κB activating oncogene in liver cancer [11]. On the other hand, another study showed that combined genetic deletion of RIPK1 and TRAF2 in liver parenchymal cells contributed to the development of hepatocellular carcinoma [15], suggesting a suppressive role of TRAF2 in HCC tumorigenesis.

In this study, we revealed mechanistically the role of TRAF2 in liver cancer cells. We found that TRAF2 is highly overexpressed in human liver cancer tissues, and high TRAF2 expression is associated with worse pathological characteristics of HCC samples, especially tumor grades as well as with poor survival of patients. Biological characterization of TRAF2 revealed that TRAF2 depletion significantly suppressed growth and survival of liver cancer cells in both in vitro cell culture and in vivo xenograft models. Taken together, our results demonstrated that TRAF2 plays a promoting role in growth and survival of liver cancer cells.

p62 was previously reported to be involved in liver disease, as p62 is a major component of the liver protein aggregates [40]. Liver-specific deletion of either Atg7 or Atg5 caused p62 accumulation and spontaneous development of liver adenoma [41, 42]. In this study, we found that p62 is highly expressed in HCC samples (Fig. S8). Although both TRAF2 and p62 are highly expressed in liver cancer tissues, the nude mice xenograft model revealed that ectopic expression of p62 alone has a weak effect on the proliferation of liver cancer cells. Rather, the effect of p62 was only reflected when in combination with TRAF2 depletion. Specifically, p62 knockdown partially abrogated growth suppression by TRAF2 depletion in both in vitro cell culture and in vivo xenograft models, thus acting as tumor-suppressing protein. A recent study also supports our discovery that p62 acted as a tumor suppressor in mice with persistent mTOR activation and defective autophagy [43]. Several previous studies [22, 44, 45] showed that p62 prompted tumor development and progression, when acting alone. Nevertheless, the status of TRAF2 was unclear in these studies. Given that p62 depletion only partially abrogated growth suppression of liver cancer cells by TRAF2 depletion, indicating the involvement of other signals, a subject for future investigation. Nevertheless, we excluded the possible involvement of NF-κB signal, since p62 depletion did not abrogate the decreased activity of NF-κB by TRAF2 depletion (Fig. S9).

Two previous studies showed that p62 is subjected to phosphorylation and p62 phosphorylation at S349 contributes to tumor growth through the disruption of the Keap1–Nrf2 complex [46, 47]. A recent study reported that under nutrient-deficient conditions, p62 undergoes acetylation at the K420 site, which enhances p62 binding to ubiquitin [48]. In this study, we found that p62 is subjected to modification via ubiquitylation by TRAF2 as a new substrate. Specifically, TRAF2 promoted p62 polyubiquitylation via the K63 linkage at K420 residue, which is subjected interestingly to lysosomal, not but proteasomal degradation. More importantly, polyubiquitylated p62 has biochemical consequence, namely activation of mTORC1 via enhanced binding with the components of mTORC1 complex, Raptor and RagC. Interestingly, previous studies have shown p62 acts as a scaffold for TRAF6, a family number of TRAF2, to promote K63-linked polyubiquitination of mTOR. TRAF6 activates mTORC1 by promoting mTOR K63-linked polyubiquitination and inhibits autophagy when amino acids are available [49]. In this study, we found that TRAF2 promotes K63-linked polyubiquitination of p62, which in turn actives mTORC1 and suppressed autophagy. Thus, our study adds another layer of complexity to the role of TRAF family in regulation of mTORC pathway. Moreover, we found that ectopic expression of FLAG-TRAF2 in TRAF2 deleted HCC cells increased the levels of phosphorylated S6K1 and decreased p62 (Fig. S10, lanes 2 vs 1), and bafilomycin A1, an inhibitor of lysosome, abrogated p62 reduction and had no effect on phosphorylated S6K1 (Fig. S10, lanes 4 vs 2 and 1), suggesting ubiquitinated p62 by TRAF2 is degraded by lysosome.

Our study strongly suggested that growth suppression induced by TRAF2 depletion is through mTORC1 inactivation in a manner dependent of p62 polyubiquitylation, since the p62 K420 mutant had much-reduced capacity of being ubiquitylated and consistently lacked ability to rescue growth suppression phenotype induced by TRAF2 depletion. Unfortunately, in clinical samples, we have not found an obvious correlation between TRAF2, p62 and p-4EBP1(Fig. S11). Although there was no statistical difference, the overall tendency was in line with our expectations. One possible reason is the insufficient clinical sample size. The future study is geared to increase the sample size to verify the correlation between TRAF2, p62 and mTOR downstream proteins.

In summary, our study supports an oncogenic role of TRAF2 in promoting growth and survival of liver cancer cells. Mechanistically, it is achieved by promoting p62 polyubiquitylation to enhance its binding with Raptor-RagC, leading to mTORC1 activation (Fig. 7I). Thus, our study validated TRAF2 as a therapeutic target for liver cancer therapy.

## Materials and methods

### Cell culture and transfection

Human liver cancer cell lines HepG2, Huh7, PLC/PRF/5, Hep3B, human embryonic kidney 293 (HEK 293, hereafter referred to as 293) cells, and mouse embryonic fibroblasts (MEFs) were saved in State Key Laboratory for Diagnosis and Treatment of Infectious Diseases, the First Affiliated Hospital, Zhejiang University School of Medicine. All cells were cultured in Dulbecco’s modified Eagle’s medium supplemented with 10% (V/V) fetal bovine serum, penicillin (100 U/ml), and streptomycin (100 U/ml). All the cells were cultured at 37 °C in a humidified incubator with 5% CO_2_. All cell lines were authenticated by short tandem repeat (STR) profiling and were tested negative for mycoplasma contamination. Transient transfected with the siRNA oligos or corresponding plasmids using Lipofectamine 3000 (Invitrogen), according to the manufacturer’s instructions, cells were analyzed 36–48 h after transfection.

### CRISPR/Cas9-mediated TRAF2 or p62 deletion HCC cell lines

Huh7 and Hep3B cells with TRAF2 or p62 deletion were generated by the CRISPR/Cas9 technology. sgRNA targeting TRAF2 or p62 was subcloned into the plasmid pSpCas9(BB)-2A-Puro (PX459). HCC cells were transfected with the plasmids and selected with puromycin for 3 days, and single clones were picked under a microscope. For each gene, we select two targeted sequences and were as follows: TRAF2-sgRNA1: 5′-CCT GCG GAG GAC GTT TCT GC-3′; TRAF2-sgRNA2:5′- CCT GCA GAA ACG TCC TCC GC-3′; p62-sgRNA1:5′- AAT GGC CAT GTC CTA CGT GA-3′; p62-sgRNA1:5′- ACG GTG GGC GGT GGT CCC GC-3′.

### Plasmids, siRNA/shRNA, and antibodies

Several tagged constructs of TRAF2 and p62 were applied in this study. FLAG-tagged plasmids were generated by cloning the corresponding cDNAs into pIRES2-EGFP vector. HA-tagged plasmids were constructed by subcloning the corresponding cDNAs into pcDNA3-HA vector. The siRNA oligos are as follows: siCtrl: 5′-ATT GTA TGC GAT CGC AGA C-3′; siTRAF2: 5′- GGA GCA TTG GCC TCA AGG A-3′; sip62: 5′-GCA TTG AAG TTG ATA TCG A-3′. Lentiviral shRNA targeting p62 was purchased from Vigene Biosciences. The following antibodies (Abs) were used: TRAF2(F-2) (Santa Cruz, sc-136999), TRAF2(Abcam, ab126758), p62 (MBL, PM045), FLAG (Sigma, F1804), FLAG (Cell Signaling Technology, 14793), HA (Roche, 11867423001), LC3 (Cell Signaling Technology, 2775), p-S6K1 (Cell Signaling Technology, 9234), S6K1 (Santa Cruz, sc-230), p-S6 (Cell Signaling Technology, 4858), S6 (Cell Signaling Technology, 2217), p-4EBP1 (Cell Signaling Technology, 2855), 4EBP1 (Cell Signaling Technology, 9452), Normal rabbit IgG (Cell Signaling Technology, 2729), mTOR (Cell Signaling Technology, 2983), RagC (Cell Signaling Technology, 3360), ACTIN (Sigma, A5441), GAPDH (Cell Signaling Technology, 2118). The plasmids used in this article were constructed in our lab, and as follows: FLAG-TRAF2, FLAG-TRAF2(1-271), FLAG-TRAF2(272-501), FLAG-TRAF2(98-501), FLAG-p62(1-266), FLAG-p62(260-345), FLAG-p62(345-440), FLAG-p62(Δ170-260), FLAG-p62K157R, FLAG-p62K295R, FLAG-p62K313R, FLAG-p62K420R, HA-TRAF2, HA-p62, HA-Ub, HA-Ub-K48R, HA-Ub-K63R, pCDNA3.1-3HA, pIRES2-EGFP.

### Immunoblotting and immunoprecipitation

For immunoblotting (IB) analysis, cells were lysed in lysis buffer (50 mM Tris pH 7.5, 1% NP-40, 0.1% SDS, 0.5% sodium deoxycholate, 0.15 M NaCl, 50 mM NaF, 1 mM EDTA, 1 mM Na3VO4, 1 mM DTT) with protease inhibitor cocktail and phosphatase inhibitors. Proteins were separated on SDS-PAGE, and then transferred to a nitrocellulose membrane. After blocking with 5% (w/v) non-fat milk, the membrane was immunoblotted with the indicated antibodies. For immunoprecipitation (IP), whole-cell extracts were incubated with the corresponding antibody for 3–5 h at 4 °C. After Protein A/G Sepharose beads addition, the incubation continued for an additional 2–4 h. To cells with exogenously expressed HA-tagged or FLAG-tagged proteins, the supernatants were incubated with bead-conjugated HA (A2095; Sigma) or bead-conjugated FLAG (A2220; Sigma) in a rotating incubator for 3 h at 4 °C. The immunoprecipitates were then washed four times with lysis buffer and analyzed by IB.

### Quantitative RT-PCR

Total RNA was isolated from cells by TRIzol reagent (Invitrogen, 15596018), then transcribed into cDNA with PrimeScript RT reagent kit (Takara, RR037A). Quantitative real-time PCR (qRT-PCR) was performed with SYBR Premix Ex Taq (Takara, RR420A) according to the manufacturer’s protocol. The primers were as follows: TRAF2(F): 5′-GCC CTT CAA CCA GAA GGT GAC-3′, TRAF2(R):5′-CCA ACC CCC AGA CAC CAG TA-3′. GAPDH(F): 5′-AGG GCA TCC TGG GCT ACA C-3′, GAPDH(R):5′-GCC AAA TTC GTT GTC ATA CCA G-3′.

### In vivo ubiquitination assay

For in vivo ubiquitylation assay, HA-Ub, FLAG-tagged TRAF2, and different indicated p62 plasmids were transfected into 293 cells. After transfection for 48 h, 293 cells were lysed in lysis buffer, the supernatants were incubated for 3 h at 4 °C, and p62 was immunoprecipitated from the cells with anti-FLAG M2 beads, followed by SDS-PAGE and IB analysis.

### Cell lysis and immunoprecipitation for mTOR complex

293T cells stably expressing FLAG-tagged proteins were collected with ice-cold PBS and lysed in lysis buffer as reported before [50]. The supernatant fractions from cell lysates were isolated by centrifugation at 13,600 rpm for 30 min. Equivalent FLAG beads were added for immunoprecipitations and incubated for 3 h at 4 °C. Immunoprecipitates were washed four times with lysis buffer, then separated on SDS-page for immunoblotting with indicated antibodies.

### Cell proliferation and colony formation assay

TRAF2 knockout HCC cells were seeded in 96-well plates in triplicate at 1500 cells per well. Cell proliferation was assessed by an ATPlite assay (6016731; Perkin-Elmer) according to the manufacturer’s instructions, and the results were obtained from three independent experiments. For cell colony formation, a total of 300 HCC cells were seeded in 60-mm dishes for 10–14 days, and then followed by Coomassie blue staining.

### Xenograft mouse model

Huh7 cells (1 × 10^6^ cells) were suspended in 100 μl PBS and injected subcutaneously into the buttock of 4–5 week old male BALB/c nude mice. The weight of the nude mice, tumor length (*L*) and width (*W*) were measured every 3 days, tumor volume (*V*) was calculated with the formula *V* = (*L* × *W*^2^) × 0.5 [51]. Animal studies were approved by the Animal Care and Use Committee of Zhejiang University.

### Human HCC samples, tissue microarray, and immunohistochemistry

Human HCC samples were obtained from The First Affiliated Hospital, Zhejiang University School of Medicine. This study was approved by Zhejiang University for Biomedical Research Ethics Committee, and all of the patients provided informed consent. 2 Human HCC tissue microarrays consisting of 180 pairs of tumors and adjacent normal tissues were obtained from Outdo Biotech Company (Shanghai, China). A total of 171 paired HCC samples were used for immunohistochemical staining and analysis of TRAF2, excluding the unqualified tissue core points. The clinical characteristics of the patients can be found listed in Supplementary Table. Immunohistochemistry (IHC) was performed in accordance with the arrays stained with indicated antibodies, followed by counterstaining with standard protocols. The slides were scanned, and the images were then digitalized for quantitative evaluation. The integrated optical density was analyzed using an immunoreactive score.

### Statistical analysis

Tumor proliferation analysis was performed using two-way repeated-measures ANOVA. Correlative analyses were performed using Pearson’s correlation coefficient to evaluate the correlation between TRAF2 and p62 expression. Survival curves were analyzed by the Kaplan-Meier log-rank test. Another statistical analysis was performed using students *t* tests, values shown are mean ± SEM. Data were carried out with three independent biological replicates. For xenograft studies, sample size (*n* = 5) are noted in figure legends. All recipient mice were age and gender-matched, therefore no formal randomization was performed. Immunohistochemistry analysis of TRAF2 and p62 were performed in blind by an expert pathologist, other experiments were not analyzed in blind. Statistical analysis was performed with the Statistical Program for Social Sciences software 28.0 (SPSS, Chicago, IL, USA) and Prism v8.01 (GraphPad Software) to compare parameters between groups. *P* < 0.05 was considered statistically significant.

## Supplementary information

supplemental materials

supplementary table

Reproducibility checklist

## Data Availability

All data needed to evaluate the conclusions in the paper are present in the paper and/or the Supplementary information. Additional data related to this paper may be requested from the authors.
