# Supplementary material for: The TRAF2-p62 axis promotes proliferation and survival of liver cancer by activating mTORC1 pathway
Source: Cell Death Differ. 2023 Apr 20;30(6):1550–62. doi: 10.1038/s41418-023-01164-7 (PMC10244464; doi:10.1038/s41418-023-01164-7)
Supplement: Supplementary file 1 — supplemental materials [file 41418_2023_1164_MOESM1_ESM.docx]

**Supplementary Materials**

**The TRAF2-p62 axis promotes proliferation and survival of**

**liver cancer by activating mTORC1 pathway**

Xue Liang, Jiping Yao, Danrui Cui, Weiyang Zheng, Yanning Liu, Guohua Lou, Bingjue Ye, Liyan Shui, Yi Sun, Yongchao Zhao, Min Zheng

**Table of content**

Fig. S1………………………………………………………………………………….2

Fig. S2………………………...………………………………………………………..3

Fig. S3……………………………………………………….………………………....4

Fig. S4……………………………………………………………………………….....5

Fig. S5……………………………………………………………….…………………7

Fig. S6…………………………………………………………………………….........9

Fig. S7…………………………………………………………………………….......11

Fig. S8…………………………………………………………………………….......13

Fig. S9…………………………………………………………………………….......14

Fig. S10..………………………………………………………………………….......15

Fig. S11..………………………………………………………………………….......16

**
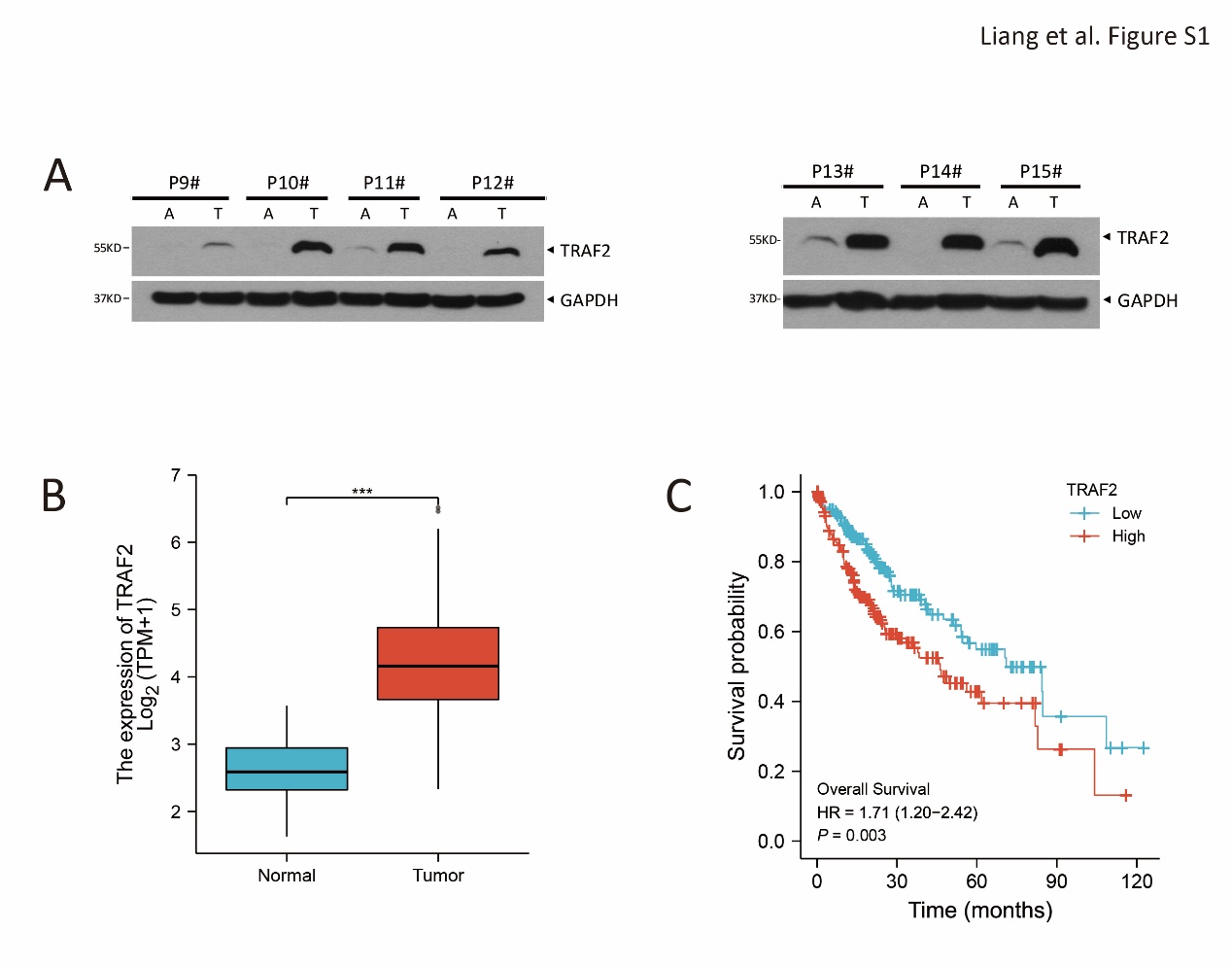
**

**Figure S1. High TRAF2 expression is associated with poor prognosis of HCC patients**

(A) TRAF2 protein level of 7 paired HCC clinical samples. (B) TRAF2 mRNA level

of HCC patients from TCGA database. ****p* <0.001. (C) Kaplan-Meier survival

analysis of patients with HCC from TCGA database. *p*=0.003.


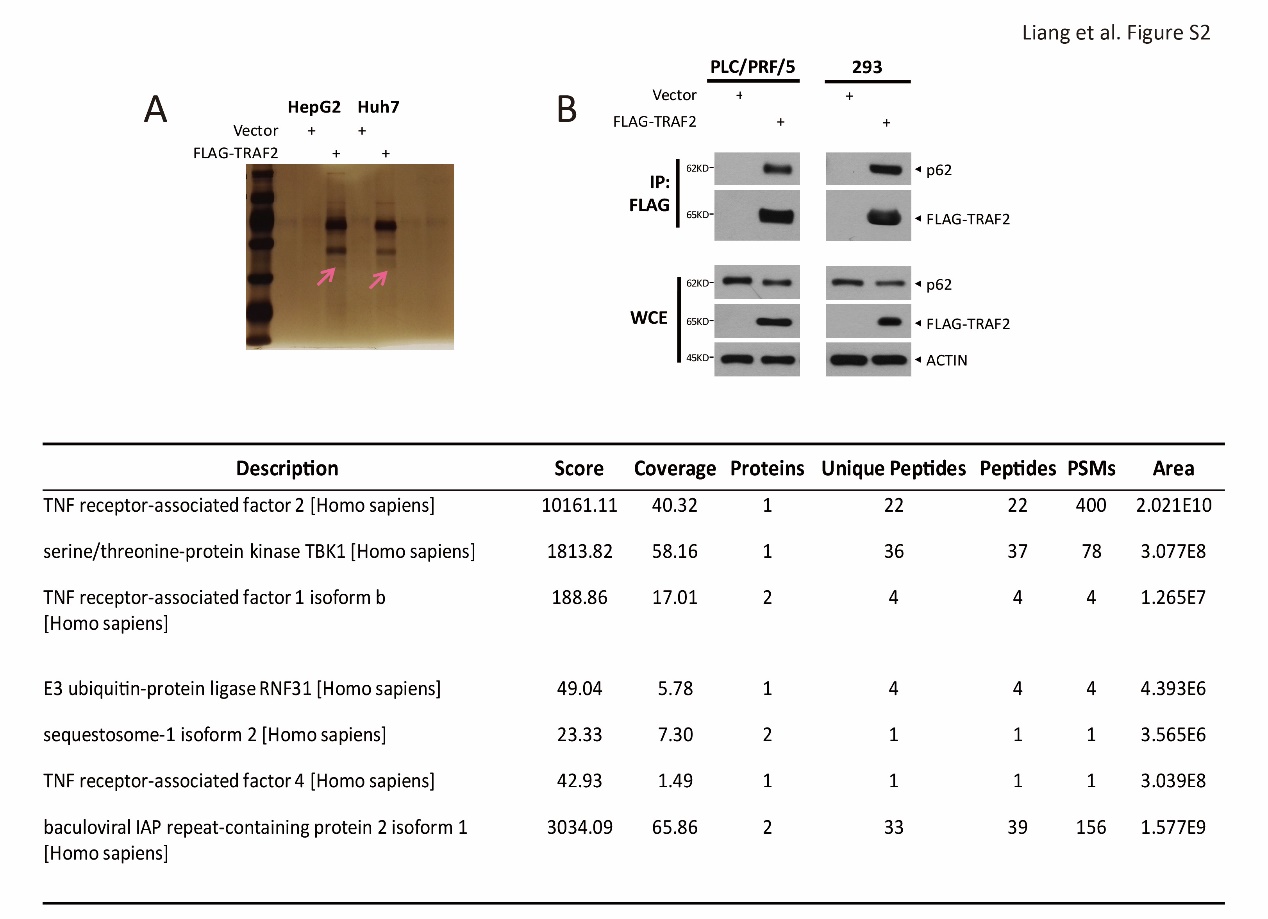


**Figure S2. TRAF2 binds to p62**

(A) Silver staining pattern of the tandem affinity purification proteins. HepG2 and Huh7 cells stably expressed vector and FLAG-TRAF2 were prepared for tandem affinity purification(up). p62/SQSTM1 was a candidate TRAF2 binding protein from mass spectrum result(below). (B) Exogenous interaction between TRAF2 and p62 in PLC/PRF/5 and 293 cells. Plasmids were transfected in PLC/PRF/5 and 293 cells, cell lysates were immunoprecipitated with agarose‐conjugated FLAG antibody, following IB with indicated antibody.


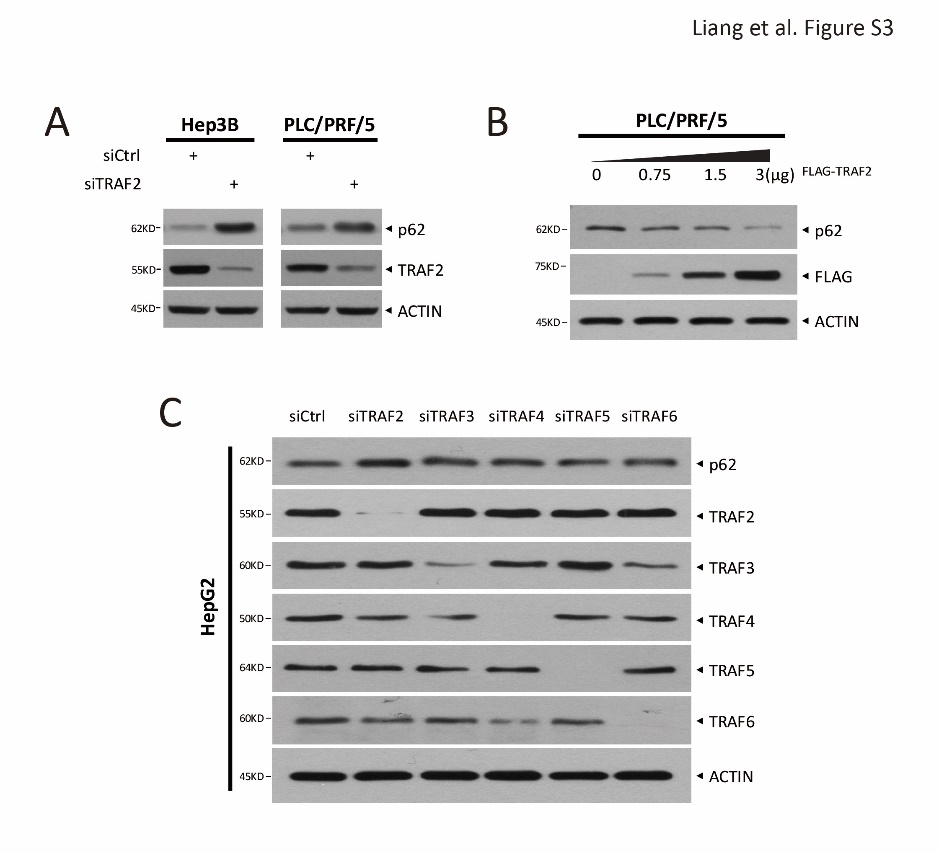


**Figure S3. TRAF2 negatively regulates the protein levels of p62 and autophagy flux**

(A) Knock down of TRAF2 increases p62 level in Hep3B and PLC/PRF/5. siCtrl and siTRAF2 were transfected in Hep3B and PLC/PRF/5 cell, and followed IB with p62, TRAF2 and ACTIN antibody. (B) TRAF2 overexpression decreases p62 level in PLC/PRF/5 cell. PLC/PRF/5 cell was transfected with increasing amounts of indicated plasmids, followed by IB with indicated Abs. (C) Compared with other TRAF2 family members, knockdown of TRAF2 significantly increased the protein level of p62. Knockdown of TRAF family members in HepG2 cell, followed IB with p62, TRAFs and ACTIN Abs.


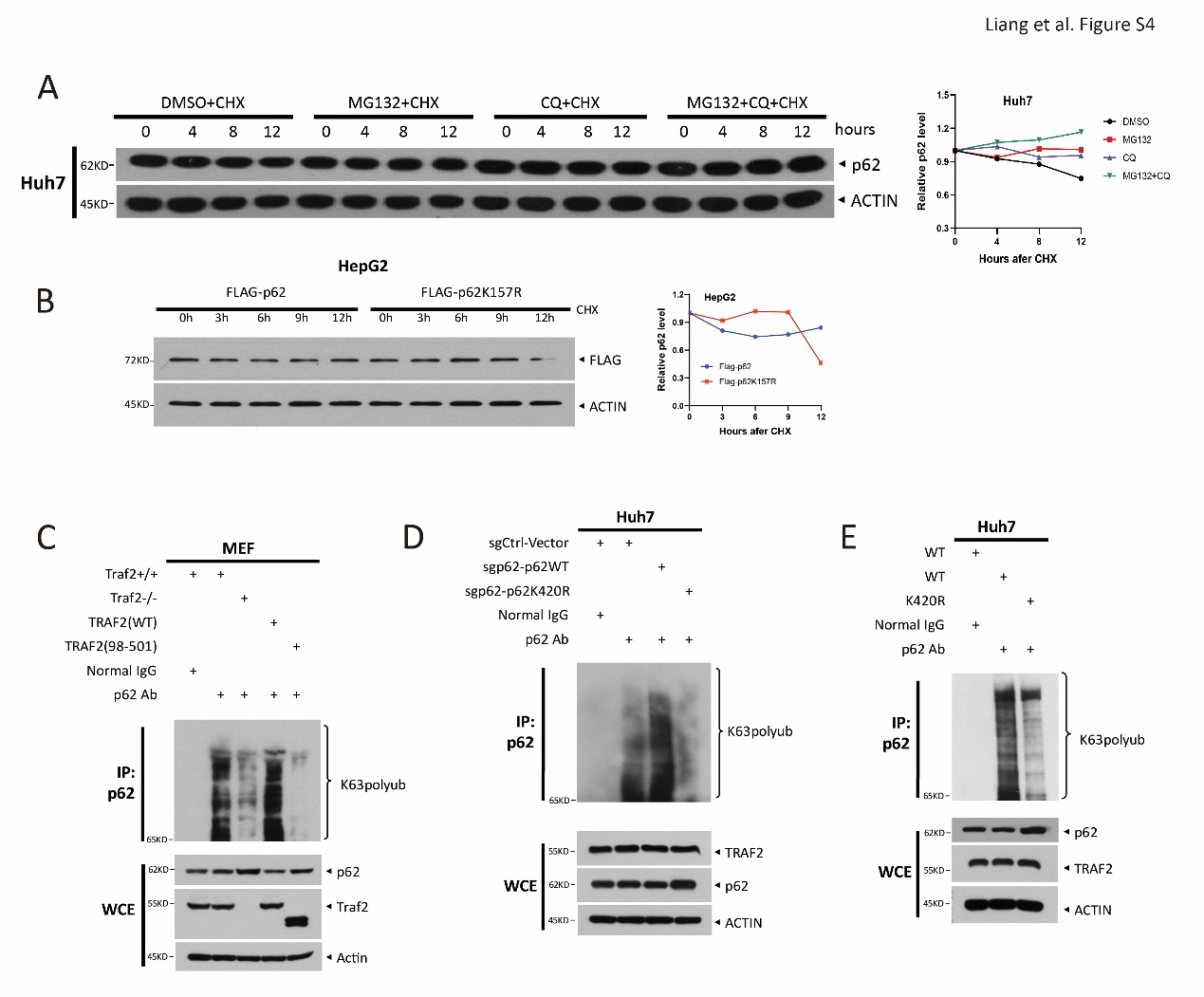


**Fig. S4. TRAF2 promotes K63-linked polyubiquitination of p62 at K420 residue and lysosome-dependent degradation**

(A) Huh7 cells with CHX (100μg/ml), were treated with MG132(20μM), CQ (50μM), and MG132 with CQ for indicated time periods, then immunoblotted with indicated Abs. (B) The half-life of p62 and the K157R mutant. HepG2 cells were transfected with FLAG-tagged p62 plasmid and K157R mutant form, then treated with CHX (100μg/ml) for indicated time, following IB with Abs. (C) TRAF2 promotes ubiquitination of p62 rely on RING domain. TRAF2 (WT), together with TRAF2 (ΔRING) mutant constructs were transfected into Traf2-/- MEF cells, followed by in vitro ubiquitination assay. (D, E) TRAF2 promotes K63 polyubiquitination of p62 at K420site. D: p62(WT) and p62K420R constructs were transfected into p62 knockout Huh7 cells, followed by in vitro ubiquitination assay. E: Ubiquitination assay in p62K420R knock-in Huh7 cell and Huh7 cell via a CRISPR/Cas9-based approach.


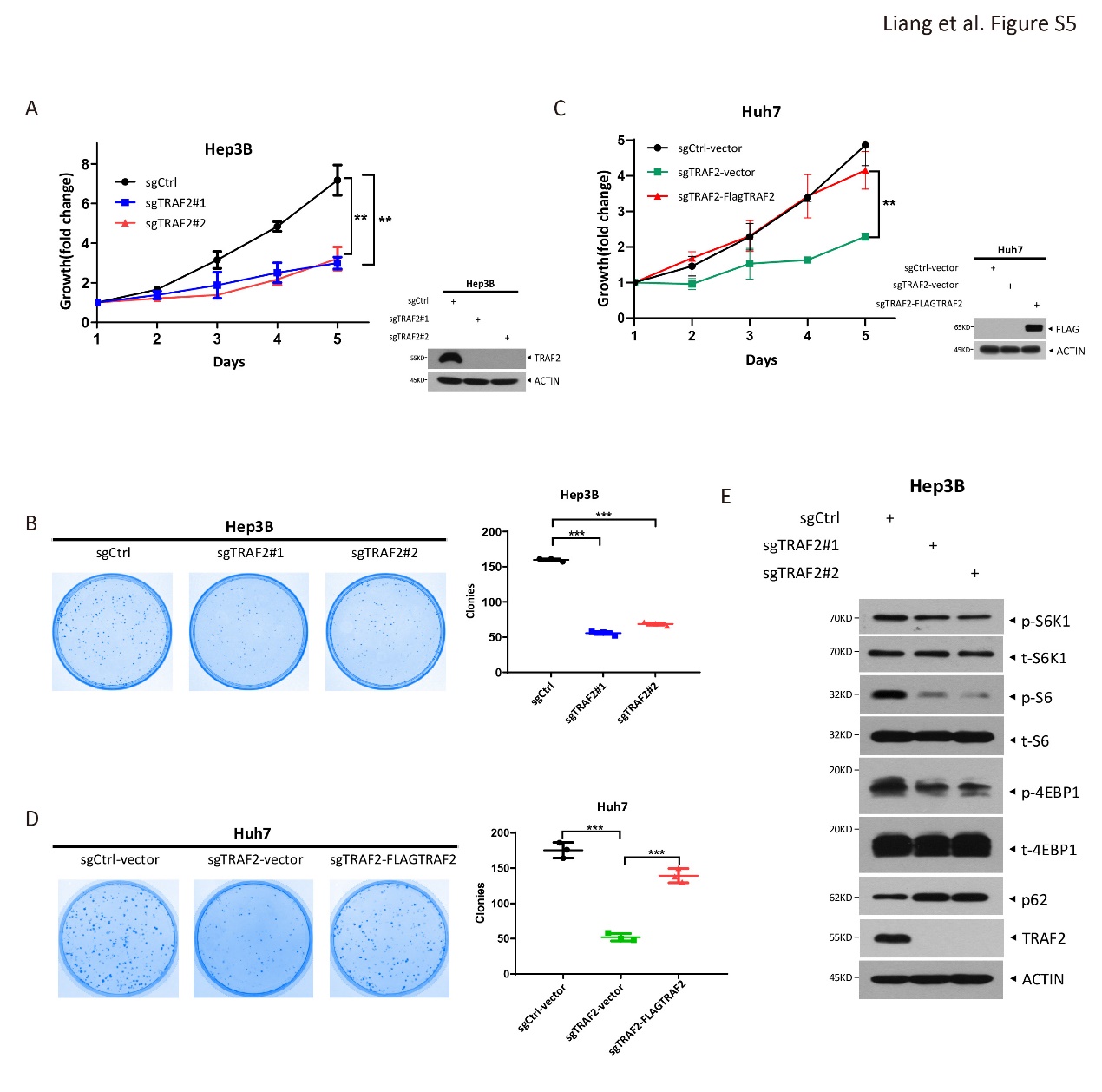


**Fig. S5. TRAF2 promotes tumor growth in vitro and in vivo by activating mTORC1**

(A) TRAF2 deletion inhibits Hep3B cell growth. Hep3B cell proliferation was determined by ATPlite cell growth assay. (B) TRAF2 deletion inhibited Hep3B cell colony formation ability. Hep3B cell clonogenic ability was expressed by coomassie blue staining (left) and colony counting (right). (C, D) Gain-of-function study showed re-transfection of TRAF2 into TRAF2-deletion Huh7 cell increased Huh7 cell growth(C) and colony formation ability(D). (E) TRAF2 deletion Hep3B cells showed decreased mTORC1 activity. Cells were lysed and followed IB with indicated Abs. Data: n=3, ANOVA analysis, ***p*<0.01, ****p*<0.001.


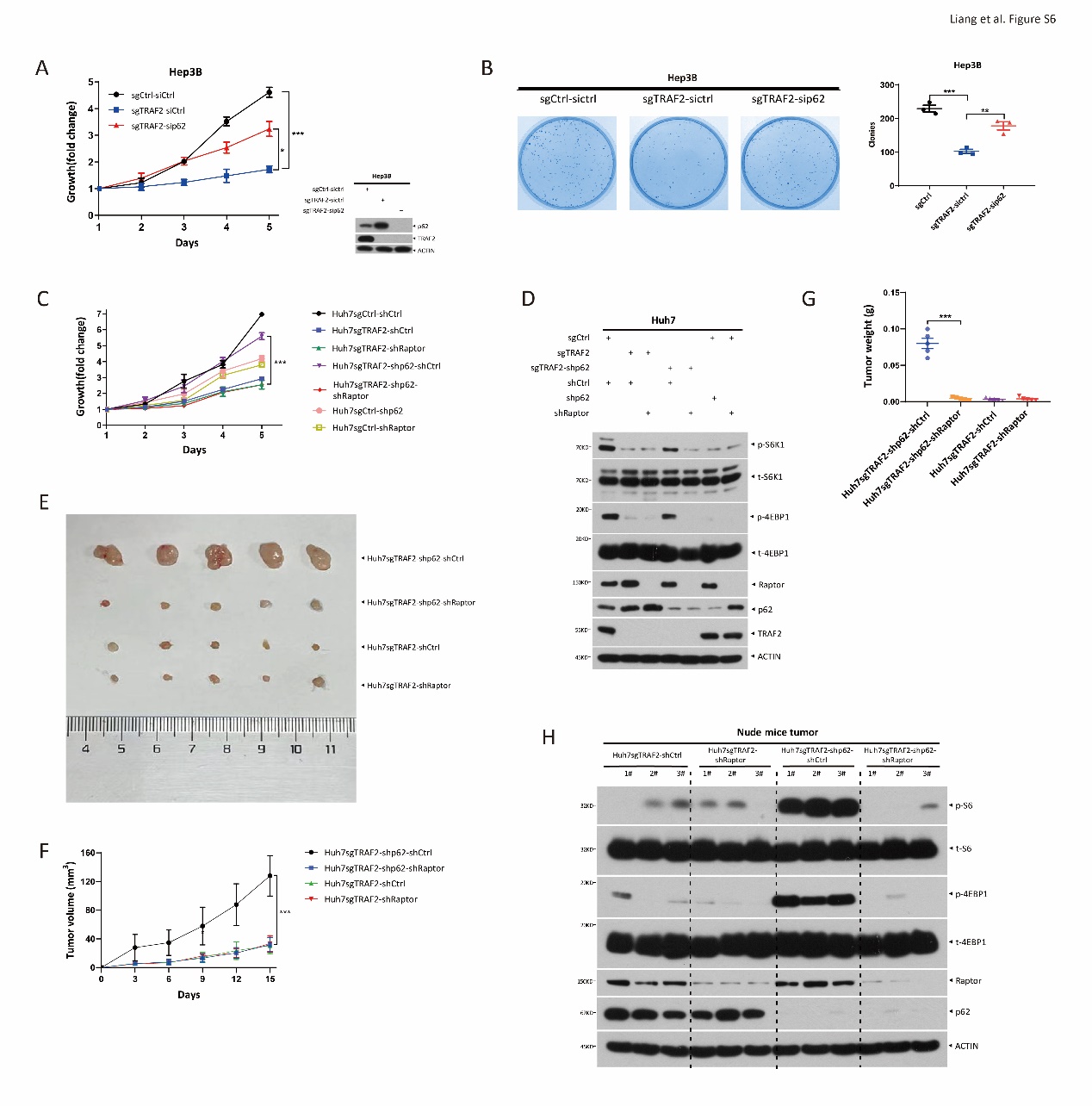


**Figure S6. TRAF2 promotes tumor growth in vitro and in vivo in a p62-dependent manner**

(A) Knock down of p62 increased TRAF2-deletion mediated Hep3B cell proliferation, cell proliferation was determined by ATPlite growth assay. (B) Knock down of p62 increased TRAF2-deletion mediated Hep3B cell clonogenic ability via coomassie blue staining (ANOVA, n=3, **P* < 0.05, ***p* < 0.01, ****p* < 0.001). (C) Cell proliferation was evaluated by the ATPlite growth assay for five days (****p*<0.001, n=3, two-way repeated-measures ANOVA analysis). (D) mTOR activation was responsible for cell growth, Huh7 cells were lysed and followed by IB with indicated Abs. (E-G) Knockdown of Raptor in Huh7sgTRAF2-shp62 cell inhibited nude mice tumor formation compared with control group, tumor volumes and weight were measured (n=5 for each group, ****p*<0.001, F: two-way repeated-measures ANOVA analysis; G: Student’s *t*-test, mean ± SEM). (H) mTOR activity was assessed of nude mice tumor, following by IB with indicated Abs.

**­
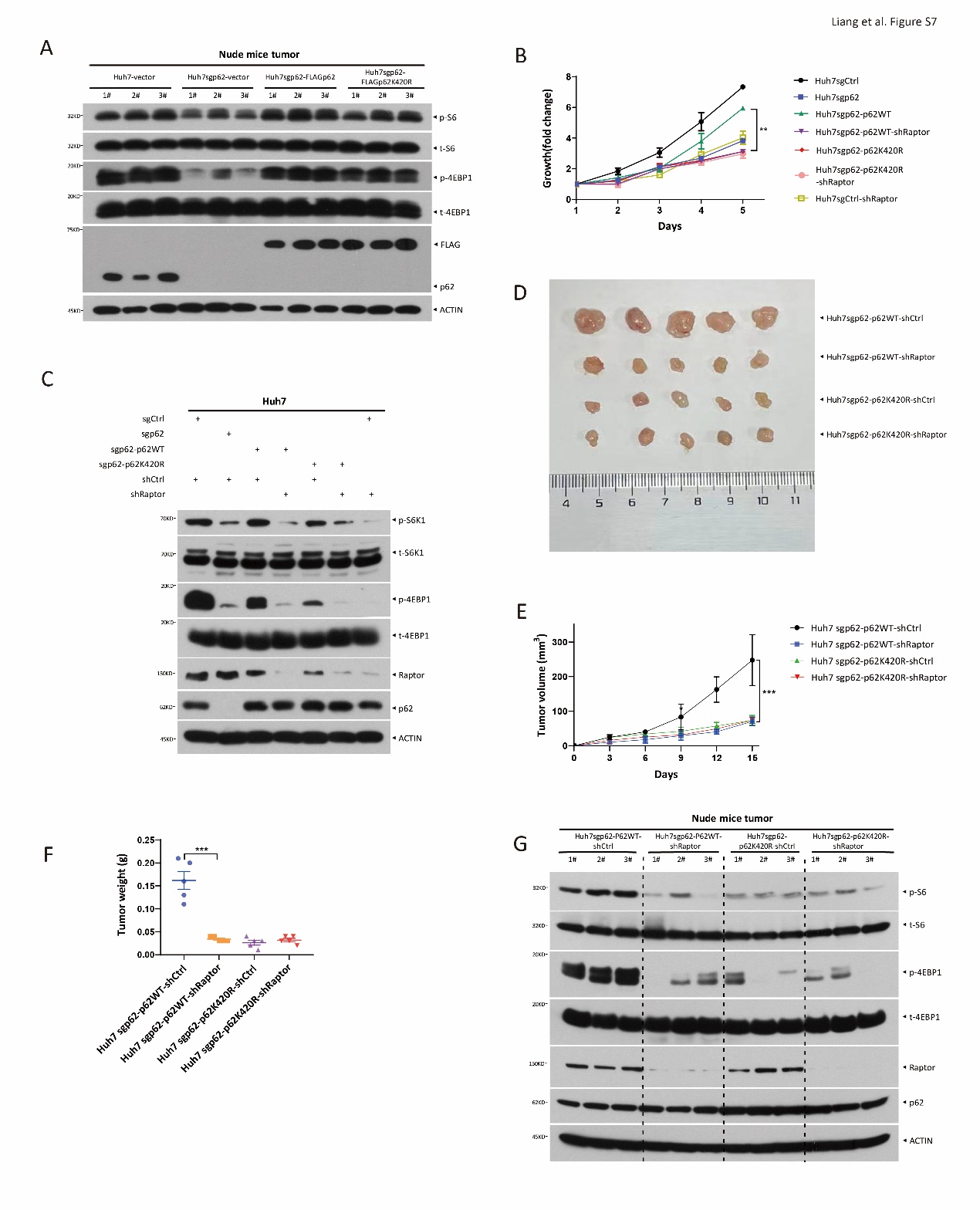
**

**Fig. S7. Ubiquitinated p62 enhances mTOR activation**

(A) mTOR activity was showed in nude mice tumor, tumor tissues were lysed and followed by IB with indicated Abs. (B) Cell proliferation was evaluated by the ATPlite growth assay for five days (****p*<0.001, n=3, two-way repeated-measures ANOVA analysis). (C) mTOR activity was showed in Huh7 cells and followed by IB with indicated Abs. (D-F) Knockdown of Raptor in Huh7-sgp62p62WT cell showed decreased nude mice tumor formation ability compared to control group, tumor volumes and weight were measured (n=5 for each group, ****p*<0.001, E: two-way repeated-measures ANOVA analysis; F: Student’s *t*-test, mean ± SEM). (H) mTOR activity was messured of nude mice tumor tissues, following by IB with indicated Abs.


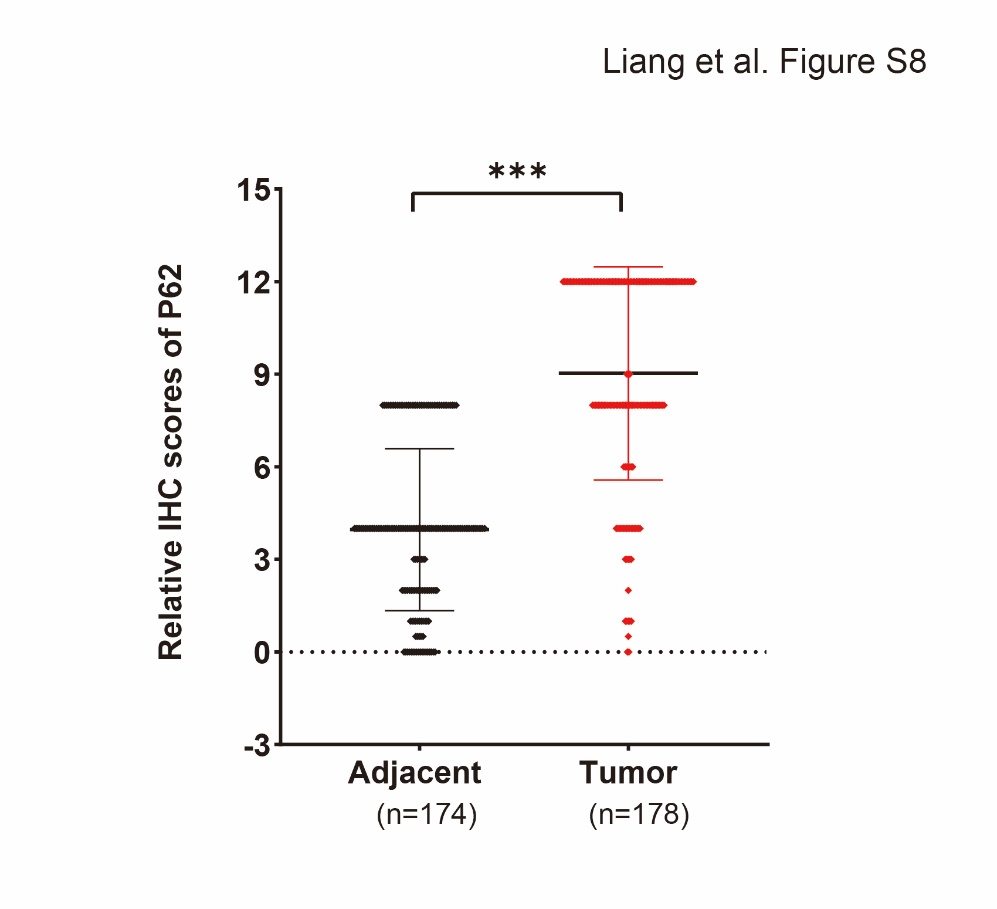


**Fig. S8. p62 was highly expressed in HCC tumor samples.**

Immunohistochemistry staining of p62 in tissue microarrays containing HCC tumor

tissues and corresponding tumor-adjacent normal tissues (****p* < 0.001, Student’s

*t*-test).


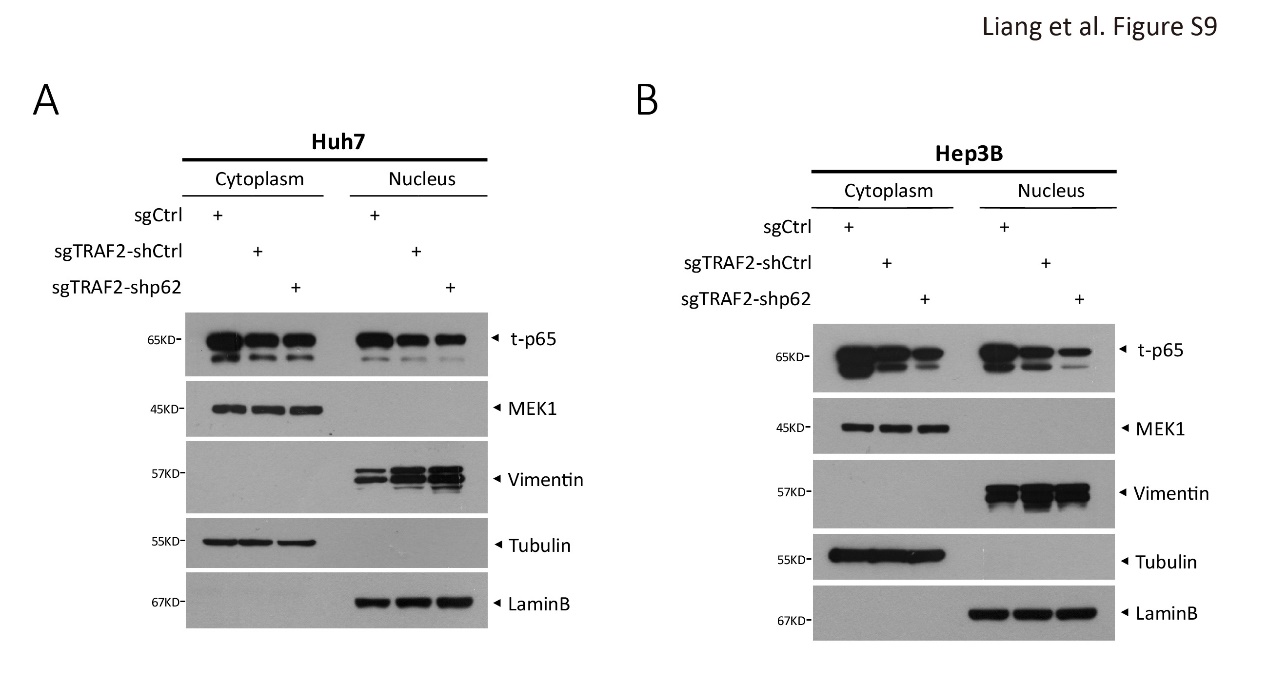


**Fig. S9. p62 depletion does not abrogate the decrease of NF-kB activity**

(A, B) Huh7 and Hep3B cells were treated with TNFα (20ng/ml) for 30mins, and then

subjected to nuclear fractionation, followed by western blotting with the indicated antibodies. Cytoplasm markers: MEK1 and Tubulin; Nuclear markers：Vimentin and LaminB.


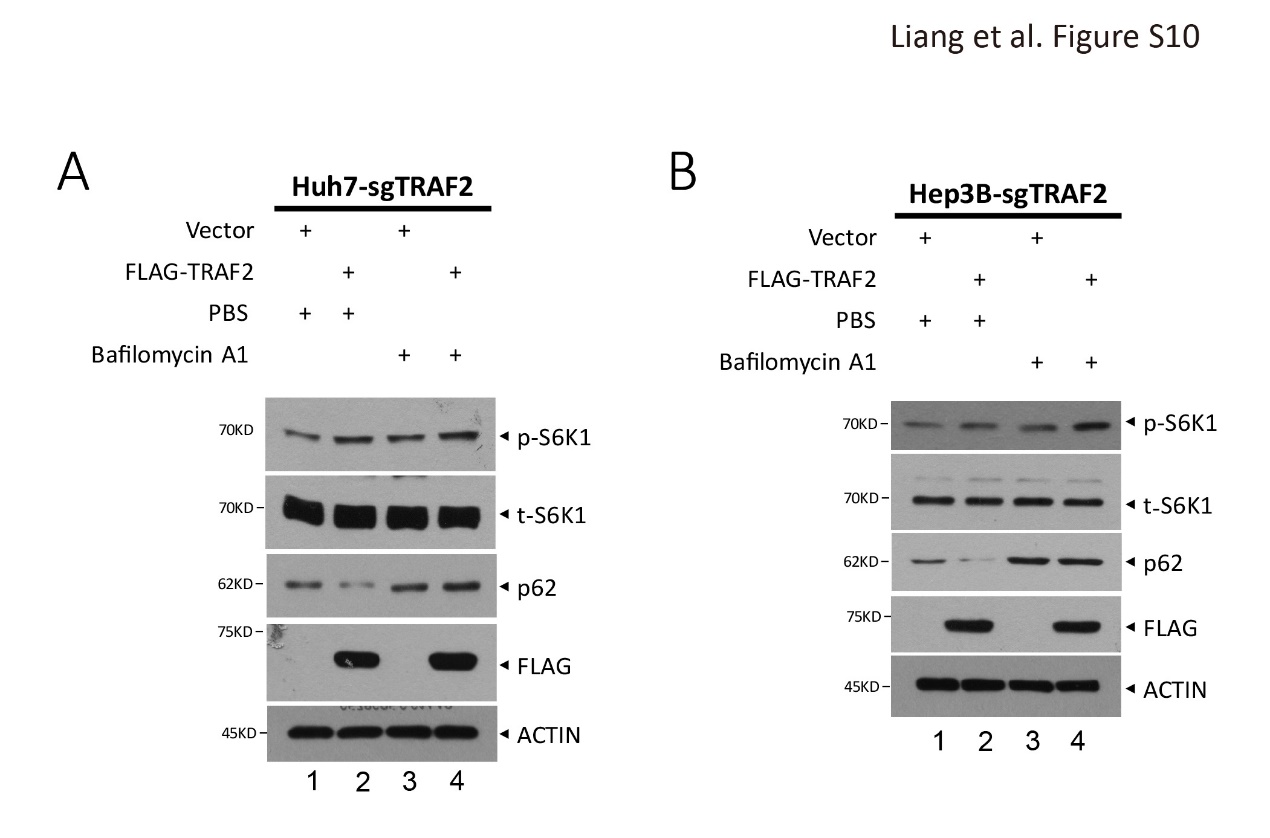


**Fig. S10. TRAF2 activates mTORC1 and promotes p62 degradation via lysosome**

(A, B) FLAG-tagged TRAF2 was ectopically expressed in TRAF2-/- Huh7 and Hep3B cells, and treated with bafilomycin A1 (50nM) for 30mins, followed by IB with indicated Abs.


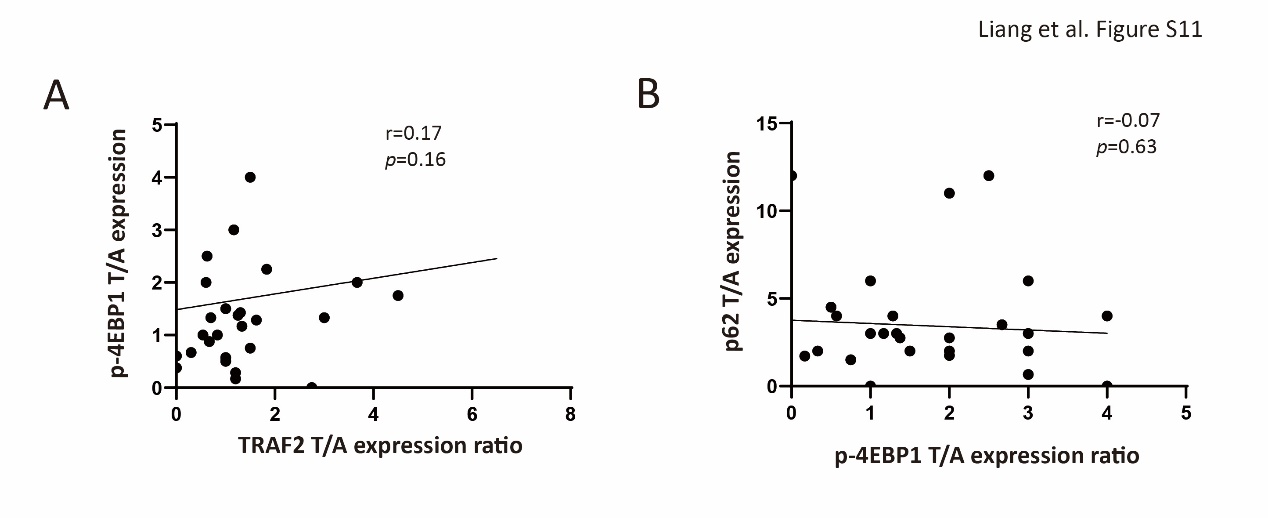


**Fig. S11. Correlation of TRAF2, p62 and p-S6, p-4EBP1 in HCC samples**

Correlation of TRAF2, p62 and p-4EBP1 in HCC tissue microarrays respectively, no significant difference was found (linear regression coefficient). T, tumor tissue. A, adjacent nontumor tissue.
