# Supplementary material for: The TRAF2-p62 axis promotes proliferation and survival of liver cancer by activating mTORC1 pathway
Source: Cell Death Differ. 2023 Apr 20;30(6):1550–62. doi: 10.1038/s41418-023-01164-7 (PMC10244464; doi:10.1038/s41418-023-01164-7)
Supplement: Supplementary file 2 — supplementary table [file 41418_2023_1164_MOESM2_ESM.docx]

**supplementary Table**

**The correlation between TRAF2 expression and clinicopathological characteristics of HCC patients**

|  | variables | TRAF2 expression | | total | χ2 | p value |
| --- | --- | --- | --- | --- | --- | --- |
|  |  | high | low |  |  |  |
| Age (year) |  |  |  |  | 0.758 | 0.384 |
|  | <55 | 61 | 43 | 104 |  |  |
|  | ＞=55 | 38 | 35 | 73 |  |  |
| Age (year) |  |  |  |  | 1.160 | 0.281 |
|  | <60 | 78 | 56 | 134 |  |  |
|  | ＞=60 | 21 | 22 | 43 |  |  |
| Sex |  |  |  |  | 0.389 | 0.533 |
|  | Female | 16 | 10 | 26 |  |  |
|  | male | 83 | 68 | 151 |  |  |
| Grade |  |  |  |  | 5.557 | 0.018 |
|  | I-II | 71 | 42 | 113 |  |  |
|  | III | 28 | 35 | 63 |  |  |
| T stage |  |  |  |  | 1.109 | 0.292 |
|  | T1 | 72 | 51 | 123 |  |  |
|  | T2-T3 | 27 | 27 | 54 |  |  |
| TNM stage |  |  |  |  | 1.109 | 0.292 |
|  | I | 72 | 51 | 123 |  |  |
|  | II-III | 27 | 27 | 54 |  |  |
| cirrhosis |  |  |  |  | 1.166 | 1.166 |
|  | negative | 14 | 7 | 21 |  |  |
|  | positive | 84 | 71 | 155 |  |  |
|  |  |  |  |  |  |  |
